# Functional organization of the HIV lipid envelope

**DOI:** 10.1038/srep34190

**Published:** 2016-09-28

**Authors:** Nerea Huarte, Pablo Carravilla, Antonio Cruz, Maier Lorizate, Jon A. Nieto-Garai, Hans-Georg Kräusslich, Jesús Pérez-Gil, Jose Requejo-Isidro, José L. Nieva

**Affiliations:** 1 Biophysics Unit (CSIC, UPV/EHU) and Department of Biochemistry and Molecular Biology, University of the Basque Country (UPV/EHU), P.O. Box 644, 48080 Bilbao, Spain; 2 Department of Biochemistry, Faculty of Biology, and Research Institute Hospital 12 de Octubre, Universidad Complutense, Madrid, Spain; 3 Department of Infectious Diseases, Virology, University Hospital Heidelberg, 69120 Heidelberg, Germany

## Abstract

The chemical composition of the human immunodeficiency virus type 1 (HIV-1) membrane is critical for fusion and entry into target cells, suggesting that preservation of a functional lipid bilayer organization may be required for efficient infection. HIV-1 acquires its envelope from the host cell plasma membrane at sites enriched in raft-type lipids. Furthermore, infectious particles display aminophospholipids on their surface, indicative of dissipation of the inter-leaflet lipid asymmetry metabolically generated at cellular membranes. By combining two-photon excited Laurdan fluorescence imaging and atomic force microscopy, we have obtained unprecedented insights into the phase state of membranes reconstituted from viral lipids (i.e., extracted from infectious HIV-1 particles), established the role played by the different specimens in the mixtures, and characterized the effects of membrane-active virucidal agents on membrane organization. In determining the molecular basis underlying lipid packing and lateral heterogeneity of the HIV-1 membrane, our results may help develop compounds with antiviral activity acting by perturbing the functional organization of the lipid envelope.

The human immunodeficiency virus type 1 (HIV-1) is an enveloped virus that follows a membrane fusion strategy to access and activate the replication cycle within the cytoplasm of host cells, typically CD4^+^ T cells and cells of the monocyte/macrophage lineage^1^. Host cell recognition and fusion with its plasma membrane is mediated by the viral envelope glycoprotein (Env)^2^. Furthermore, the physical properties and chemical composition of the HIV membrane play a key role in the entry process^3^,^4^. While much is known about the mechanistic and structural details of Env-mediated fusion^5^,^6^, the functional organization of membrane lipids is less well understood.

HIV-1 acquires its lipid envelope and Env proteins by assembly and budding at the plasma membrane of the infected cell^7^,^8^. Assembly is driven by the viral Gag polyproteins, and appears to occur in specific membrane microdomains^7^,^8^. Advanced mass spectrometry combined with protocols optimized for the isolation of viral lipids enabled the determination of the HIV-1 membrane composition^9^,^10^,^11^. These studies showed that cholesterol (Chol) and sphingomyelin (SM) are enriched in the virus at concentrations similar to those in detergent-resistant membranes^9^. Quantitative analyses also revealed that, as compared to the bulk plasma membrane, the viral membrane appears to be enriched in specific lipids, including aminophospholipids, dihydrosphingomyelin (DHSM), plasmenyl phosphoethanolamine (or plasmalogen phosphatidylethanolamine, pl-PE), phosphoinositides, or ceramide, and this composition varied depending on the producer cell lines^10^,^11^,^12^.

In addition, mature virions have been reported to expose phosphatidylserine (PS) and phosphatidylethanolamine (PE) on their external surface^13^,^14^,^15^,^16^,^17^. Thus, the membrane acquired by HIV from infected cells appears to have lost the asymmetric lipid distribution generated at the plasma membrane by ATP-dependent aminophospholipid translocases. It has been suggested that PS exposure on virions and virally infected cells may enable viruses to evade immune recognition and diminish inflammatory responses to infection^14^,^18^. Aminophospholipids may also act as cofactors for HIV-1 infection of macrophages^13^, a phenomenon possibly related to the functional modulation of viral membrane lipid packing and lateral organization^3^,^4^.

To gain insight into the molecular basis governing packing order and lateral heterogeneity in the complex HIV membrane, we have studied Giant Unilamellar Vesicles (GUVs) and monolayers made of lipids extracted from infectious HIV-1^12^. We have quantified lipid packing in the HIV-membrane GUVs by applying modified two-photon Laurdan fluorescence microscopy, and analyzed the topographic features of the monolayers by atomic force microscopy. We have compared first these complex membranes with compositionally simple models, identifying individual species that are critical for the maintenance of high order and lateral demixing. Subsequently, to assess the functional relevance of lipid order and miscibility, we have characterized the perturbing effects exerted by membrane-active agents on membranes made from virus-derived lipids, correlating with their virucidal activity.

Our results indicate that the complex HIV membrane is highly packed, but less ordered than the rigid domains segregated in DOPC:SM:Chol-based models, and may display de-mixed nanoscopic lipid assemblies modulated by the lipid composition. Fluidification and induction of nanodomain coalescence by membrane-active compounds appear to be detrimental for viral entry, and changing membrane order may thus represent an alternative target for antiviral development.

## Results

### Membrane lipid packing in HIV-1 mixtures and surrogates

**Figure 1:**
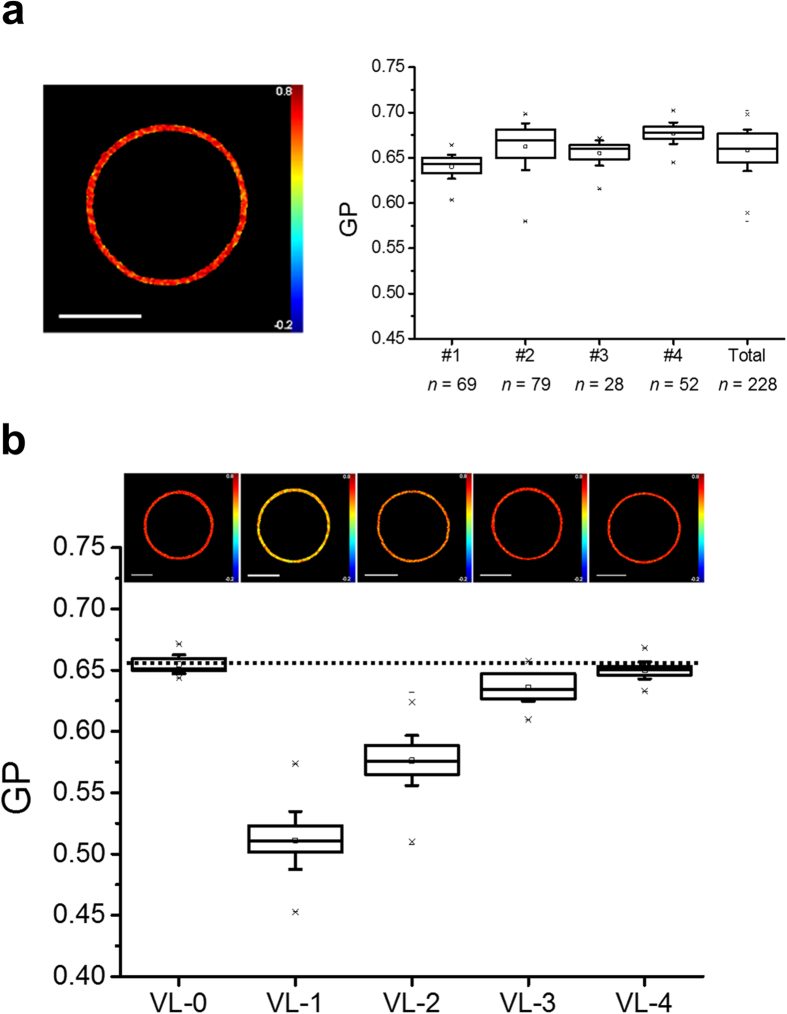
Lateral packing of HIV membrane using two-photon excitation fluorescence microscopy of Laurdan-labeled GUVs. (**a**) Laurdan GP imaging of a single HIV-reconstituted GUV (left), and GP quantification for 4 different HIV-reconstituted GUV preparations (right, #1–4). “Total” corresponds to the grouping of all data measured in the 4 preparations. GP value distributions were determined for single vesicles in a sample of size *n* as indicated, and mean values depicted as box-and-whisker plots (the ends of the whiskers represent standard deviations). (**b**) GP images and average values determined for viral-like membrane surrogates (top and bottom, respectively). The dotted line shows the mean GP value calculated for the HIV membrane (“Total” in the previous panel). Scale Bars correspond to 5 μm in all micrographs; GP color scale limits: −0.2, 0.8. GUV lipid-compositions are summarized in Supplementary Table S1.

To quantitatively determine HIV membrane packing, we performed two-photon imaging of Laurdan-stained GUVs made from a complex mixture of lipids extracted from purified, infectious HIV-1 (Fig. 1). Laurdan is a hydrophobic probe highly sensitive to solvent polarity. Its fluorescent emission undergoes a large spectral shift due to the reorientation of solvent polar molecules in the fluorophore vicinity during the time it spends in its excited state^19^,^20^. When incorporated in a lipid bilayer, Laurdan’s spectral shift is independent of the polar head charge and is, instead, a function of the hydration and viscosity of the membrane, reflecting its phase state. A wavelength-ratiometric parameter called Generalized Polarization (GP) quantifies Laurdan’s emission spectral shift from tightly packed bilayers (hardly hydrated) to loosely packed (highly hydrated) membranes, thus providing an indirect measurement of membrane order through packing. Laurdan GP imaging of GUVs allows direct visualization of phase-separated lipid domains, although its quantification is prone to artifacts and requires exquisite adjustment of the optical set-up. Very small deviations from a circularly polarized excitation beam result in a systematic uncertainty on the determination of the GP. Supplementary Fig. S1 illustrates this effect and our approach to identify and correct small polarization mismatches, thereby allowing accurate GP quantification in the complete equatorial section of the vesicle.

Figure 1a displays a ratiometric GP image of a GUV made of HIV lipids (micrograph on the left) and the average GP values determined for 4 different preparations (right panel). All reconstituted HIV membranes displayed a single macroscopic ordered phase with mean average GP values ranging between 0.64 ± 0.01 and 0.68 ± 0.01. The mean value measured for the complete population (*n* = 228) was 0.66 ± 0.02. By comparison, the 1,2-dioleoyl-sn-glycero-3-phosphocholine DOPC:SM:Chol (2:2:1) mixture (henceforth Ld/Lo phase-separated model) laterally separated in two domains: one with a GP value of 0.70 ± 0.02, corresponding to a liquid-ordered (Lo) phase, defined as enriched in saturated sphingolipids and cholesterol in a highly condensed state, and the other with a GP value 0.06 ± 0.06, corresponding to a liquid-disordered (Ld) phase, enriched in unsaturated glycerophospholipids in a disordered state (Supplementary Fig. S2a). These observations were consistent with an overall high degree of lipid packing in the viral envelope. However, the significant differences in the distribution of the collected GP values (Supplementary Fig. S2) further suggest that the lipid packing level of the viral membrane is below that measured in the Lo domain of the phase-separated model, or in the two-component SM:Chol (1:1) mixture (0.71 ± 0.01, Supplementary Fig. S2b).

To identify the HIV lipid species responsible for sustaining lipid packing, Laurdan GP values were further determined for GUVs made from synthetic virus-like (VL) mixtures (Fig. 1b and Supplementary Table S1). Models combining 5 lipids (VL-2 and VL-3) have been derived from the HIV lipidome established by Brügger *et al.*^9^ and are commonly employed as surrogates of the viral membrane^21^,^22^,^23^. Similarly to the HIV-1 mixture, all VL mixtures displayed a single macroscopic phase (GP images on top). However, quantification of the GP values revealed significant differences in lipid packing (bottom panel).

The simpler models VL-0 and VL-1 combine a single glycerophospholipid, DOPC with SM and Chol. VL-0 matches the GP value of the HIV membrane using a similar Chol content (ca. 45%), and DOPC and SM content as determined from the GP-phase diagram of the DOPC:SM:Chol ternary mixture^24^ (Supplementary Fig. S3). Thus, VL-0 embodies a simplified model of the HIV membrane displaying a comparable level of lipid packing. In contrast, the VL-1 mixture combines DOPC with the mole fractions of SM and Chol found in the viral lipidome^9^,^10^,^11^. The Laurdan GP value determined for the VL-1 mixture was much lower than for GUVs made from virus-extracted lipids or VL-0. The lipid mole ratios reveal that it is the higher SM content which sustains the higher GP value of VL-0 in comparison to the VL-1 mixture. The GP values displayed in Fig. 1b demonstrate that an increase of mixture complexity to render VL-2, VL-3 and VL-4 surrogates, may emulate the ordering effect of SM.

The GP value of the VL-1 mixture increased significantly when replacing a fraction of DOPC by the aminophospholipids DOPE and DOPS (VL-2 model^21^). A further increase of the GP value was observed for the VL-3 mixture containing 1-palmitoyl-2-oleoyl phospholipids (POPC; POPE; POPS) instead of the respective di-oleyl lipids. Each monounsaturated glycerophospholipid separately contributed to increased lipid packing (Supplementary Fig. S4a). A subtle increase of the Laurdan GP value was finally observed for GUVs made of the VL-4 mixture. In this case, fractions of POPE and SM were replaced with pl-PE and DHSM, respectively, two species that account for a significant mole percentage of virion lipids^9^. GUVs made of this 7-lipid mixture exhibited a membrane order degree comparable to that measured for GUVs made of HIV-1 lipid extracts (dotted, reference line).

**Figure 2:**
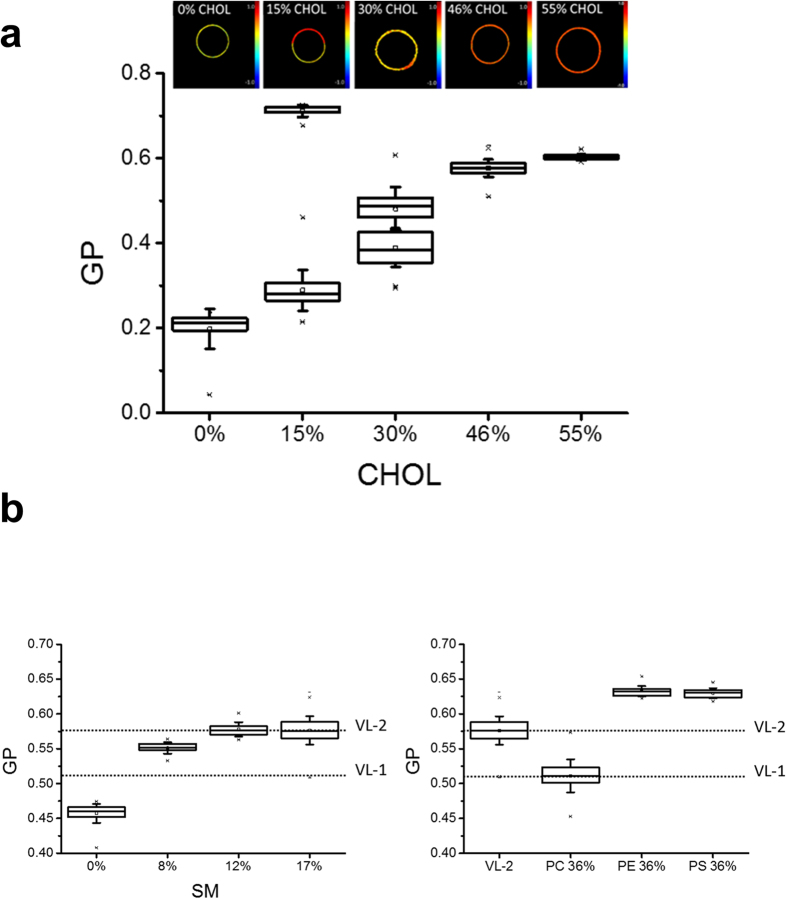
Membrane lateral packing dependence on the lipid components of viral mimic VL-2. (**a**) GP images (top) and average determination (bottom) with increasing amounts of Chol. Two GP values are defined for the laterally separated lipid compositions containing 15 and 30 mol % Chol, the upper one corresponds to the more ordered/dehydrated domain and the lower one to the more disordered/hydrated domain. GP color scale limits: −1.0, 1.0. (**b**) Membrane order dependence on phospholipid species, SM (left) and PC, PE and PS (right). Chol content was 46 mol % in all cases. The dotted lines indicate the average GP for VL-1 and VL-2 mixtures. Conditions otherwise as in Fig. 1.

The higher Laurdan GP values of increasingly complex mixtures indicated that HIV lipid packing primarily depends on polar head-group identity and acyl-chain saturation degree, while the presence of DHSM and pl-PE may also contribute to increase the order level as previously suggested^9^. Laurdan GP values displayed in Fig. 2 further illustrate the contribution to the lipid packing of single components in the generally used VL-2 model. As expected, Chol is the main determinant of membrane order (Fig. 2a). The VL-2 mixture devoid of Chol displayed GP values consistent with a single fluid phase. Inclusion of 15–30 mole % of Chol induced macroscopic Lo-Ld domain separation, while increasing its content to reach the levels existing in the viral envelope generated a single, highly ordered phase. With 15 mol % Chol the lipid packing difference between the ordered and disordered phases (ΔGP) was less pronounced than in the Lo-Ld separation model (ΔGPs of 0.42 and 0.64, respectively, compare Fig. 2a and Supplementary Fig. S2), while this difference became even smaller with 30 mol % Chol (ΔGP = 0.09). These findings are consistent with recent reports indicating that subtle compositional variations may lead to a variety of lipid packing states in model and natural membranes^25^,^26^.

Figure 2b shows the effects of the three phospholipids SM, PE and PS, when PC was used as reference. SM enhanced the order level in a dose-dependent way, and above that measured for the VL-1 ternary mixture (left panel). Interestingly, SM levels below those existing in the mixture were sufficient to attain maximal order. In line with the composition-dependent lipid packing, both, PE and PS, increased GP (right panel, see also Supplementary Figs S4b and S4c). These observations suggest that aminophospholipids may take part in regulating the ordered vs. disordered balance in de-mixed fluid domains^27^. They also imply that the loss of inter-leaflet asymmetry in virions emerging from infected cells does not necessarily result into more loosely packed lipid bilayers as previously suggested^13^.

### Miscibility of the HIV membrane lipids

**Figure 3:**
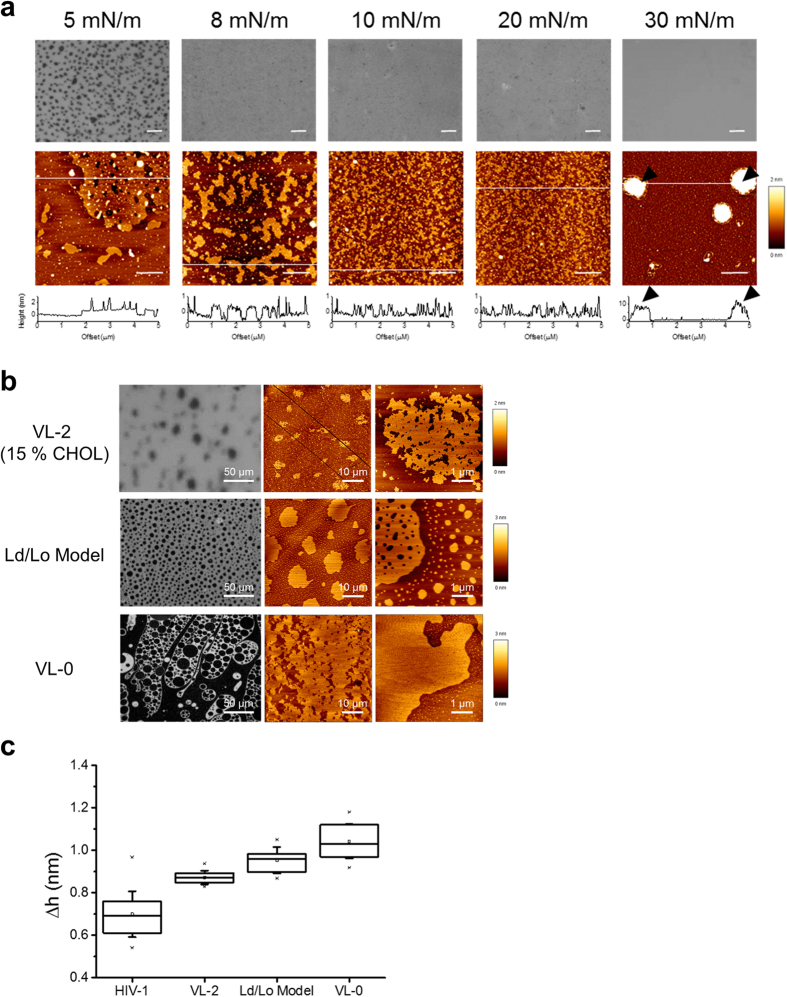
Liquid-liquid immiscibility of the HIV membrane. (**a**) Epifluorescence microscopy (top) and AFM (bottom) images taken from a HIV mixture film compressed at the indicated surface pressures. In epifluorescence images, the bright background (liquid-disordered) corresponds to the fluorescence emission of the Rho-DOPE probe that gets excluded from the ordered domains (dark spots only observable at Π = 5 mN/m). Plots below AFM images display the height profiles for the trajectories indicated by the white lines. Monolayer collapse at Π = 30 mN/m is evidenced from the accumulation of material excluded from the interface (arrowheads mark white spots with heights of ca. 10 nm). Scale bars are 100 and 1 μm in epifluorescence and AFM images, respectively. (**b**) Lateral organization of films made of the VL-2 mixture containing 15% of Chol (mol:mol), the DOPC:SM:Chol (2:2:1) phase-separated model, or the VL-0 surrogate (top, center and bottom, respectively). Epifluorescence and AFM images were obtained at fixed Π = 8 mN/m. Laterally de-mixed micron-size domains can be discerned by both techniques (left and center panels). At higher amplification AFM images disclose the different morphologies of the clusters (rightmost column). Other experimental conditions were as described for the previous panel. (**c**) Height difference between de-mixed domains and the surrounding lipid surface.

To determine the liquid-liquid immiscibility of the HIV membrane (resulting from the differences in packing and dynamics of lipids in different liquid phases), Langmuir–Blodgett films compressed to defined surface pressures (Π) were transferred onto mica and analyzed by combining epifluorescence imaging and Atomic Force Microscopy (AFM) as previously described^28^. Figure 3a shows the miscibility transitions and topographic information of HIV-1 lipid films as a function of Π at both the microscopic and the nanoscopic lengthscales (top and bottom panels, respectively). The monolayer collapses at 30 mN/m, as evidenced by the partial exclusion of some material away from the interface (arrowheads). At the lower Π values of 20 and 10 mN/m the epifluorescence micrographs display homogeneous films, while the AFM analysis reveals a coarse texture and the presence of de-mixed nanoscopic domains. At 8 mN/m, strip-shaped domains with a rough morphology of the boundaries can be better discerned in the AFM images. Further lowering Π to 5 mN/m results in the coalescence of those nanodomains into larger clusters that can also be observed in the epifluorescence images as dark (ordered) domains in a fluorescent (disordered) background. Thus, the 5–8 mN/m threshold seems to represent a miscibility transition pressure for the HIV mixture, in accordance with similar values reported for ternary mixtures containing Chol^29^.

Differences in lateral organization for the HIV membrane model and the Ld/Lo phase-separated model are suggested by the comparative epifluorescence/AFM analysis displayed in Fig. 3b. Low magnification images obtained at a fixed Π = 8 mN/m displayed micron-sized, round domains in films made of the VL-2 mixture containing 15% Chol and in films of the DOPC:SM:Chol-based phase-separated and VL-0 models (top, center and bottom panels, respectively). Analyzing these films with AFM, however, which provides nanoscale resolution, revealed rough domain boundaries for the micron-size platforms in the VL-2 (15% Chol) mixture. In contrast, segregated domains in the ternary DOPC:SM:Chol mixtures exhibited smooth contours, as previously reported to occur in Ld/Lo phase-separated model systems^30^,^31^.

The very small size and rough morphology of the boundaries in rigid clusters of the HIV mixture are consistent with the low line tension at domain interfaces that characterize lipid systems undergoing fluctuations in phase composition^32^. This coarse pattern is maintained at surface pressures below collapse of the monolayer, which emphasizes its relevance for the envelope organization. In contrast, the VL-0 surrogate displays an ordered background that contains laterally separated large platforms with smooth shapes. Thus, for comparable lipid packing degrees (identical GP values), the HIV lipid membrane and the simple VL-0 surrogate display different degrees of complexity regarding the lateral organization at the nanoscopic level. This suggests that model membranes based on 3-lipid combinations are not optimal surrogates of the HIV membrane.

As further shown in Fig. 3c, the height difference of the rigid domains relative to their surrounding fluid environment (Δh) correlated with their size and morphology (Fig. 3a,b). Line tension at the rims of de-mixing phases intensifies as Δh increases^33^, thereby sustaining larger ordered domains in the VL-2 (15% Chol) sample than in the HIV mixture. Even higher Δh values were observed in the DOPC:SM:Chol Ld/Lo phase-separation model and in the VL-0 surrogate (Fig. 3c), indicative of higher interfacial energy in these samples, which might in turn explain the separation into perimeter-minimizing, smoothly shaped domains.

### Alteration of HIV membrane lipid packing and miscibility by membrane-active agents

**Figure 4:**
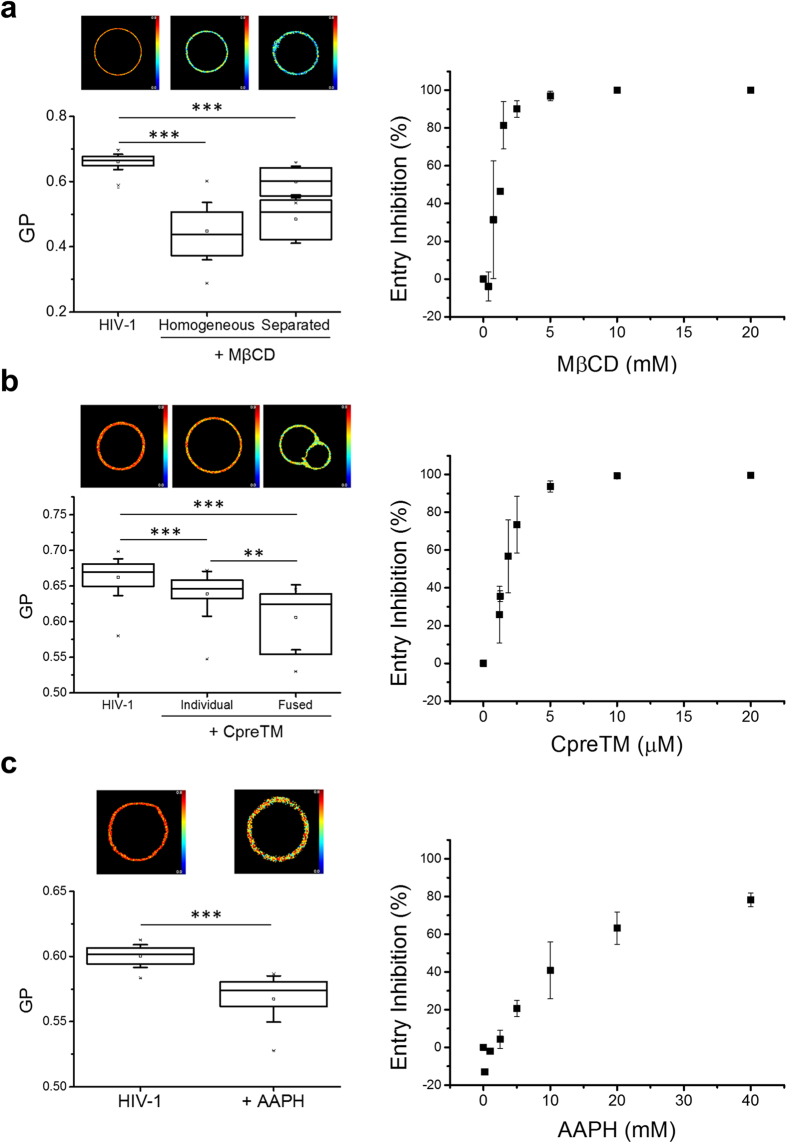
Effect of membrane-active compounds on HIV membrane lateral packing (left panels) and pseudovirus cell entry (right panels). (**a**) Left: Samples were incubated with M*β*CD (1 mM) for 15 minutes before collecting GP images from Laurdan-labeled individual GUVs. The group of vesicles that displayed better fitting to two-component distributions is plotted using two boxes, the upper one corresponding to the more ordered domains and the lower one to the more disordered regions. Right: HIV-1 pseudoviruses were pre-attached to lysine-coated plates^57^ and treated with increasing concentrations of M*β*CD. After washing, reporter TZM-bl cells were layered on top, and entry (gene transduction) inferred from the number of total cells expressing GFP, as described previously^39^. Values represent means ± SD of three independent assays. (**b**) Samples were treated with CpreTM peptide. Average GP values were determined for GUVs treated for at least 15 minutes with 1 μM peptide (left). The population of GUVs displaying membrane diaphragms were sorted out and measured separately. (**c**) Samples incubated with AAPH. GP values were determined after 15 minutes incubation with 20 mM AAPH (left). Conditions otherwise as in the previous panels. (***p < 0.0001; **p < 0.001). GP color scale limits: 0, 0.9.

**Figure 5:**
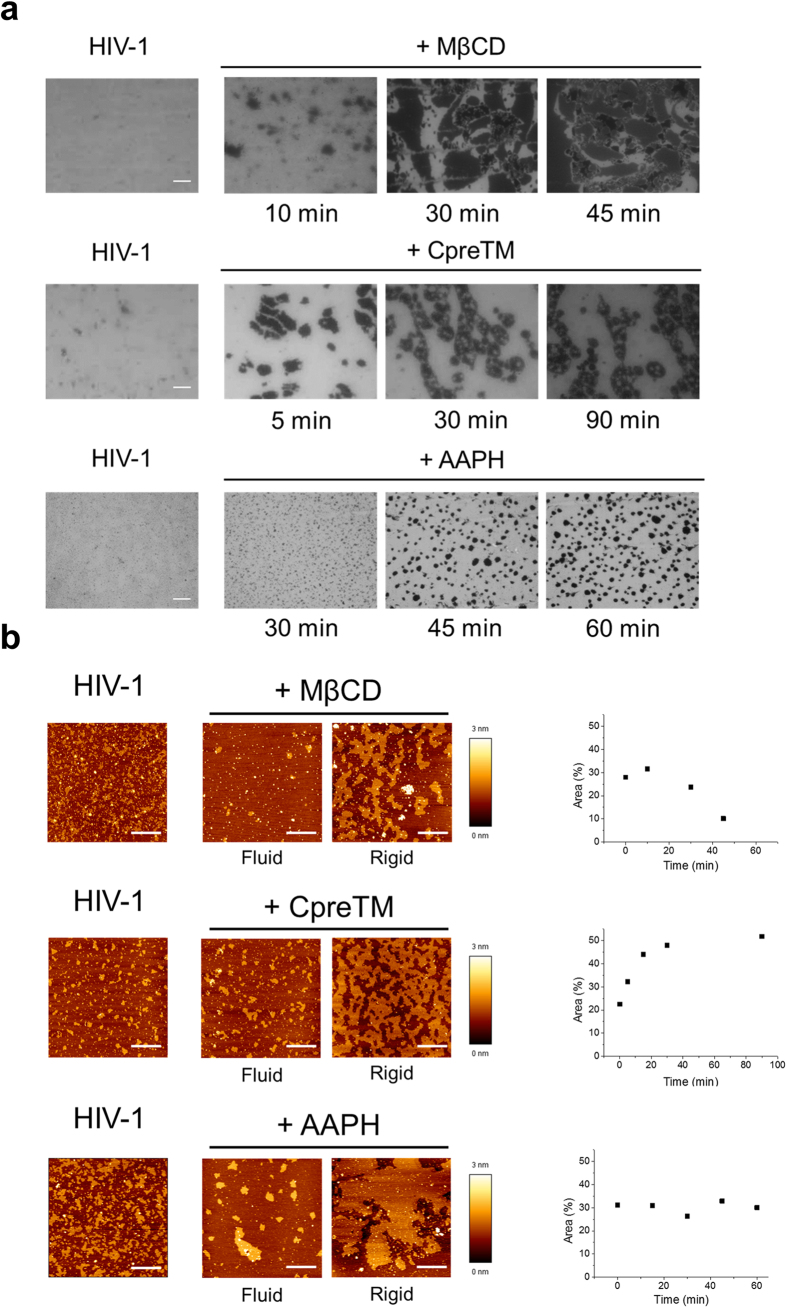
Effect of membrane-active compounds on the lipid miscibility of the HIV membrane. (**a**) Epifluorescence images of HIV monolayers showing the effects of M*β*CD (1 mM), CpreTM (0.1 μM) or AAPH (5 mM) as a function of the incubation time (top, middle and bottom panels, respectively). (**b**) Nanoscopic structure of disordered-like and condensed areas as determined by AFM analyses of the virucide-treated HIV monolayers. Panels on the right depict the change in the area percentage covered by ordered domains as a function of the incubation time. Conditions otherwise as in the previous Fig. 4b.

To establish the functional relevance of the lipid packing and miscibility states accessible to the natural HIV membrane, reconstituted GUVs and lipid monolayers were treated with membrane-active compounds displaying virucidal activity (Figs 4 and 5). Figure 4 (left panels) compiles the effects exerted on HIV lipid packing by three different compounds, namely: the Chol-extracting compound methyl-β-cyclodextrin (M*β*CD)^34^,^35^, the membranolytic peptide CpreTM^36^ and the peroxyl radical generator 2,2′-Azobis(2-methyl-propionamidine) dihydrochloride (AAPH)^37^. Cell entry assays were carried out in parallel to confirm the efficacy of these compounds as virucides (right panels).

Laurdan-labeled HIV GUVs treated for at least 15 minutes with M*β*CD exhibited lower Laurdan GP values than untreated controls, indicating a decrease in lipid packing induced by this compound (Fig. 4a, right), which was consistent with the effect observed in Laurdan-stained virus particles^12^. At the end time-points of incubation, homogeneous phase GUVs with a low GP value co-existed with vesicles displaying lateral separation of more fluid and less fluid domains (see Supplementary Fig. S5 for an example of the latter). These findings give further support to the intrinsic capacity of the complex HIV membrane to access a variety of lipid packing states, which evolve modulated by the Chol content as in the surrogate mixtures (see previous Fig. 2a). Emphasizing the functional relevance of this capacity, Chol extraction by M*β*CD blocks virus-cell entry^34^,^35^,^38^ (Fig. 4a, left).

Membranolytic, aromatic-rich, short peptides such as C5A from hepatitis C virus or CpreTM from HIV, both showing antiviral activity against HIV^36^,^39^, encompass an additional class of membrane-active virucidal agents^40^. As shown in Fig. 4b (left), Laurdan-labeled HIV GUVs treated with this peptide displayed a subtle, but significant reduction of the GP values. In these samples, the lowest GP values were measured for aggregated vesicles displaying a contact diaphragm, consistent with a correlation between membrane-perturbing and fluidifying effects induced by the peptide. Again, the membrane-fluidifying CpreTM was virucidal for HIV (Fig. 4b, right).

Lipid oxidative damage also perturbs the HIV membrane and suppresses infectivity^41^,^42^. The effects of HIV lipid oxidation on lipid packing and cell entry were studied after exposure of HIV GUVs and pseudovirus particles to the free radical initiator AAPH^37^. Reduction of the Laurdan GP value indicated an overall fluidification of the membrane as a consequence of its oxidative action (Fig. 4c, left), while the pseudovirus exposure to AAPH inhibited cell entry (Fig. 4c, right). Contrasting our findings, the oxidative action of some compounds has previously been correlated with increased lipid packing and reduced membrane fluidity^43^, effects postulated to impair viral fusion^42^. To ensure that the fluidification effect observed in our samples did not pertain uniquely to the complex HIV mixture, Laurdan GP values were also calculated and averaged in AAPH-treated VL-2 and VL-4 single GUVs. As shown in the Supplementary Fig. S6, the GP values of VL-2 GUVs decreased readily upon incubation with AAPH, while more stringent conditions were required to observe the same fluidifying effect in the case of the more packed VL-4 surrogate. To prove that lipids following our treatment were subject to similar degradative processes, we also determined Laurdan GP values for vesicles made of the same lipid composition used in previous studies (i.e., POPC)^43^. In this case the apparent discrepancy disappears as we observe higher GP values consistent with an increase in lipid packing of AAPH-treated POPC vesicles. We surmise that the single-lipid POPC model does not reproduce the variety of lipid packing states inherent to the complex HIV membrane.

Together, the observed changes in mean Laurdan GP values induced by the different virucidal agents underpin the correlation between decrease in HIV lipid packing (related to membrane fluidification) and antiviral activity. In other words, keeping a high level of envelope lipid packing seems to be crucial for the virus entry function.

The effects of the membrane-perturbing compounds on the in-plane organization of the HIV mixture were evaluated next (Fig. 5 and Supplementary Figs S7–S9). Epifluorescence images of monolayers treated with these compounds exhibited lateral de-mixing into micron-size domains (Fig. 5a), which were not observed in parallel, untreated controls. The morphological alterations undergone by those domains differed in the three samples. Round domains appeared at early times of incubation with M*β*CD (top panels). These domains disaggregated and mixed with the surrounding membrane at later times, as evidenced in experiments using Rho-DOPE and Topfluor-Chol probes to label the fluid (red) and ordered (green) domains, respectively (Supplementary Fig. S7). The double-label experiments also revealed the appearance of dark patches, consistent with the exclusion and/or extraction from membrane areas of the fluorescent probes. CpreTM, on the other hand, induced the formation of a network of ordered domains, disclosing embedded circular patches of disordered lipid regions at the longest times of incubation (middle panels). Finally, AAPH treatment resulted in the appearance of round rigid domains interspersed in the fluid background that remained stable over time (bottom panels).

Higher resolution AFM of fluid and ordered areas provided additional insights into the underlying mechanisms of virucide-induced de-mixing of fluid domains (Fig. 5b and Supplementary Fig. S8). The AFM images of M*β*CD-treated samples revealed depletion of rigid nanodomains from fluid regions, and appearance of large clusters of nanodomains within the ordered regions (Fig. 5b, top-left). In the latter regions nanodomains compacted over time until they started to extrude their contents into the surrounding membrane (Supplementary Fig. S8a). Consistently, the overall amount of membrane surface covered by the ordered (thicker) phase decreased at longer times of incubation (Fig. 5b, top-right). Thus, the observed effects are consistent with Chol depletion by M*β*CD, first occurring in the more disordered phase, which becomes more fluid and expands increasing rigid nanodomain propensity to cluster. After equilibration, Chol is also removed from the more ordered regions, which results in domain disaggregation (Supplementary Figs S7 and S8a).

By comparison, the CpreTM peptide did not affect the nanoscopic organization of the more fluid areas (Fig. 5b middle-left), but induced a significant increase of the rigid phase area (Fig. 5b middle-right and Supplementary Fig. S8b). In this case, the peptide seems to generate ordered patches within the fluid phase, fixing the pre-existing rigid domains into a continuous web that becomes more compact over time (Supplementary Fig. S8b). The appearance of circular fluid phase domains within these rigid platforms further suggests that they expand radially^44^, concomitant with merger and compaction. Thus, the peptide reduced the overall membrane order (Fig. 4b), but increased the rigid phase area (Fig. 5b). We hypothesize that this counterintuitive observation can be explained by the induction of membrane restructuring and lipid packing loosening in the vicinity of the peptide (see our previous work^45^). These local perturbations could also induce rigid domain growth when evolving within phase boundaries, thereby affecting membrane organization at a larger scale.

Lastly, fluid areas in AAPH-treated films appeared to be depleted of separated rigid domains. The rigid domains again aggregated into ordered micron-size domains (Fig. 5b bottom-left and Supplementary Fig. S8c). Based on the effects observed for PC:SM:Chol monolayers^46^, it was argued that oxidized glycerophospholipids stabilize micron-sized domain separation by increasing the hydrophobic mismatch between Lo and Ld phases. The chemical modification is expected to make the Ld phase thinner resulting in augmented line tension at inter-domain boundaries and thus promoting the coalescence of rigid domains into energy-minimizing larger platforms^46^. The oxidized HIV mixture may follow this behavior, as inferred from the changes in Δh values observed upon incubation with AAPH (Supplementary Fig. S9). Consistent with Ld phase thinning, the height difference (Δh) increased with time and reached a maximum of ca. 1.1 nm concomitant with the stabilization of the separated domains. Interestingly however, this process appears to drive coalescence of rigid domains, but does not promote their formation, since the surface covered by the rigid phase remained constant (Fig. 5b bottom-right).

Overall, the monolayer results displayed in Fig. 5 support that, regardless of the mechanism, induction of lateral de-mixing correlates with virucidal activity. This implies that regulation of liquid-liquid immiscibility plays a functional role in the viral entry process.

## Discussion

Cryoelectron microscopy of virions reveals that a lipid membrane covers most of the HIV-1 surface in contact with the external medium^47^. Compared to the plasma membrane of producing cells, this lipid bilayer is enriched in Chol and aminophospholipids PS and PE^9^, which all appear to be accessible on the external leaflet^3^,^13^,^17^. Several findings support that these selective features are of functional relevance: (i) Chol-chelating/depleting compounds inhibit HIV fusion and infectivity^3^,^38^; (ii) Interaction with T-cell immunoglobulin (Ig) and mucin domain (TIM) proteins tether HIV-1 particles bearing surface-exposed PS to the cell surface as well as to each other, thereby blocking release from infected cells^40^; (iii) the chimeric antibody, bavituximab, used to identify and target PS exposed at membrane surfaces, suppresses productive HIV infection^14^; and (iv) cyclotide peptides that associate with membranes through specific binding to PE, exert virucidal activity on HIV virions^16^,^17^.

To gain insight into the molecular basis underlying the functional organization of the lipid HIV membrane, we have carried out a detailed study of the properties of GUVs made from infectious HIV-1 or from synthetic lipid mixtures regarding membrane order and domain miscibility. We have first produced homogeneous populations of compositionally complex GUVs enriched in Chol^24^,^48^ (Figs 1 and 2), and accurately quantified lipid packing at the membrane (Supplementary Fig. S1). Ratiometric imaging revealed a high level of HIV membrane order (Fig. 1a), which was lower than that observed for the segregated Lo phase in DOPC:SM:Chol GUVs (Supplementary Fig. S2).

We have found that comparable levels of lipid packing can be attained by increasing SM content in the three-lipid, simplified model VL-0 (Fig. 1b and Supplementary Fig. S3). The ordering effect of SM can however be reproduced by incorporating aminophospholipids and saturated acyl chains to render more complex 5- and 7-lipid VL-2, VL-3 and VL-4 surrogates (Fig. 1b and Supplementary Fig. S4). Topographic AFM analyses of HIV membrane monolayers reveal a coarse pattern, including de-mixed rigid nanodomains (Fig. 3a). Such pattern was not observed in Lo/Ld phase-separated or simplified VL-0 models (Fig. 3b). Thus, the HIV membrane composition appears to be set to optimize high levels of lipid packing and ensure lateral de-mixing. While highly packed membranes can contribute to stabilize the structure of the viral particle in the external medium, lateral separation into rigid nanodomains might be required for the cell entry function (see below).

Taken together, (i) the high membrane order (Fig. 1), (ii) the small ΔGP-s between ordered and disordered regions regulated by Chol (Fig. 2), (iii) the presence of de-mixed nanoscopic rigid domains (Fig. 3) and (iv) the broken line-boundaries separating rigid clusters from the surrounding membrane (Fig. 3), strongly suggest that the HIV membrane is actually set at the threshold of fluid phase de-mixing^32^. Consistently, small, but significant changes in lipid packing induced by membrane-active compounds (Fig. 4 and Supplementary Figs S5 and S6) strongly affected the lateral organization of the HIV membrane as inferred from changes in rigid nanodomain clustering and line tension observed by AFM (Fig. 5, and Supplementary Figs S7–S10). Further underpinning the biological relevance of HIV-1 lipid packing and de-mixing states, the observed structural changes of the virus-derived membrane correlated with functional inhibition of pseudovirus by the membrane-active compounds.

**Figure 6:**
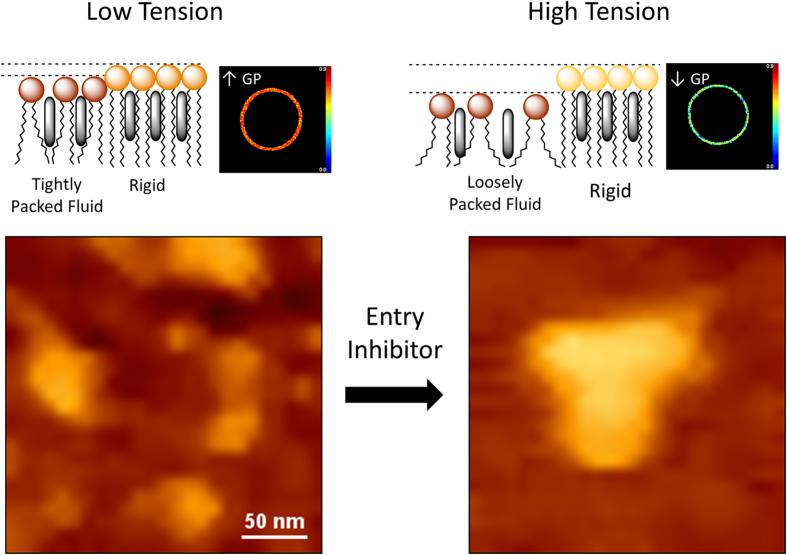
Model for the functional organization of the HIV membrane. Left: competence for entry depends on the presence of a highly packed fluid state and low line tension (top), which allows separation of small, convoluted rigid nanodomains (bottom). Membrane fusion would evolve more efficiently at the boundaries between those nanodomains and surrounding membrane^52^. Right: fluidification and/or thinning of the more disordered phase increases line tension (top) and promotes domain clustering (bottom). Coalescence results in a reduction of the separated domain perimeter available for membrane fusion. Compounds that reduce lipid packing could induce the transition between both states, and hence act as inhibitors of the entry function. AFM images covering a surface that approximates that of the viral lipid envelope correspond to untreated (left), and AAPH-treated viral lipid mixtures (right). The lighter color of the demixed domain in the latter sample denotes a larger height difference over the surrounding membrane.

The use of membrane-targeting virucidal compounds, altering the properties of the viral lipid membrane, has been proposed as a viable alternative for the development of broad-spectrum virus entry inhibitors^42^,^49^,^50^. Our data indicate that compounds that perturb the phase state of the HIV membrane by increasing fluidity or inducing ordered domain clustering might encompass effective virucides. Thus, in contrast to previous reports suggesting that virucides act by rigidifying the viral membrane^42^, our data indicate that fluidification of the membrane may be detrimental for infectivity. We suggest that a highly packed fluid phase (i.e., enriched in Chol) is a prerequisite for retaining the nanoscopic sizes and convoluted perimeters of de-mixed rigid domains (Fig. 6). Reducing packing of the fluid phase may alter the size and clustering of rigid nanodomains and change the overall lipid miscibility in a way that could be incompatible with its function in viral entry (Fig. 6).

In its original form, the membrane raft hypothesis stated that molecular assemblies of lipids, laterally separated under a variety of physiological conditions, play a functional role in cell compartmentalization of trafficking and signaling^51^. A recent model proposes an additional role in HIV fusion, which would occur at boundaries between disordered and ordered domains in both, target cell and viral membrane^23^,^52^. Our data provides evidence for the nanoseparation of the viral membrane, showing that lateral discontinuities may indeed exist in the highly ordered viral membrane, which opposes the view of the HIV lipid envelope as a laterally homogeneous Lo-like membrane. We have observed that fluidification of the more disordered regions and, hence, induction of ordered domain clustering (model in Fig. 6), interferes with viral entry. We speculate that both fluidification and clustering may affect Env function following two non-exclusive mechanisms: (i) larger rigid clusters may trap functional Env peplomers in distant areas of the membrane and hinder their lateral diffusion to the sites of fusion^47^,^53^,^54^, (ii) merging of small nanodomains into large clusters could reduce the ratio of boundary lipids to total domain lipids, which would diminish the influence of boundaries upon fusion^52^.

In conclusion, the use of HIV-reconstituted membranes has allowed us to gain extraordinary knowledge on the viral membrane at the molecular level. In this sense, the studied biophysical model systems and the reported effects could constitute valuable approaches with biomedical applications such as drug screening. Nonetheless, definitive transfer of our observations to the functional viral particles awaits the future implementation of optical methodologies allowing, precise, nanoscale phase measurements.

## Materials and Methods

### Materials

1,2-dioleoyl-*sn*-glycero-3-phosphatidylcholine (DOPC), 1-palmitoyl-2-oleoylphosphatidylcholine (POPC), 1,2-dioleoyl-*sn*-glycero-3-phosphatidylethanolamine (DOPE), 1-palmitoyl-2-oleoylphosphatidylethanolamine (POPE), 1,2-dioleoyl-*sn*-glycero-3- phosphatidylserine (DOPS), 1-palmitoyl-2-oleoylphosphatidylserine (POPS), cholesterol (Chol), egg sphingomyelin (SM, containing ≈ 86% N-palmitoyl SM), dodecanoyl dihydrosphingomyelin (DHSM), 1-(1Z-octadecenyl)-2-oleoyl-*sn*-glycero-3-phosphatidylethanolamine (pl-PE), 23-(dipyrrometheneboron difluoride)-24- norcholesterol (Topflour-Chol) and N-(lissamine rhodamine B sulfonyl)-1,2-dioleoyl-*sn*-glycero-3-phosphoethanolamine (Rho-DOPE) were purchased from Avanti Polar Lipids (Birmingham, AL, USA). 6-dodecanoyl-2-dimethylaminonaphthalene (Laurdan) was obtained from Molecular Probes (Eugene, OR, USA). Phospholipid stock concentrations were determined by phosphate assay. 2,2′-Azobis(2-methyl-propionamidine) dihydrochloride (AAPH) and Methyl-β-cyclodextrin (MβCD) were purchased from Sigma-Aldrich (St. Louis, MO). The CpreTM peptide used in this study was synthesized in C-terminal carboxamide form by solid-phase methods using Fmoc chemistry, purified by reverse-phase high-performance liquid chromatography (HPLC), and characterized by matrix-assisted laser desorption ionization–time-of-flight (MALDI-TOF) mass spectrometry (purity > 95%).

### Virus purification and lipid extraction

Lipid extraction from infectious particles was performed as described^12^, according to the method of Bligh and Dyer^55^. The HIV-1 NL4–3 virus strain was harvested from cocultures of infected and uninfected MT-4 cells before cytopathic effects were observed. Particles were concentrated from cleared media by centrifugation through a cushion of 20% (wt:wt) sucrose in 150 mM NaCl, 10 mM Hepes, pH 7.4 and further purified by velocity gradient centrifugation on an Optiprep gradient (Axis-Shield, Oslo, Norway).

### Preparation of giant unilamellar vesicles

Giant unilamellar vesicles (GUVs) were produced by spontaneous swelling of lipid films deposited on 40 μm silica beads as described^24^,^48^. Briefly, lipid-Laurdan mixtures (0.125 mg total lipid) in CHCl_3_:CH_3_OH (9:1) were dried in a vacuum desiccator for 1 h to remove the organic solvent. The desiccated lipid was hydrated for 1 h at temperatures above the transition temperature of the mixture (typically 55 or 65 °C for the mixtures with highest SM content) and subsequently subjected to 30 cycles of extrusion through two 0.4 μm pore size polycarbonate filters in an Avanti Mini Extruder at 65 °C. Five microliters of silica beads was then mixed with 20 μL of the LUV suspension, separated in ≈3 μL drops on a teflon film, and vacuum-dried for 45 min. Dried beads covered with lipid were collected and transferred to a 7.5 g/L sucrose buffer to induce spontaneous swelling of GUVs. Finally vesicles were transferred to the observation dish in an isosmotic 10 mM HEPES, 150 mM KCl (pH 7.4) buffer.

### Multiphoton Fluorescence Microscopy

Images were acquired on a Leica TCS SP5 II microscope (Leica Microsystems GmbH, Wetzlar, Germany). For multiphoton imaging the sample was excited at 780 nm using a femtosecond-pulsed titanium-sapphire Mai-Tai Deepsee laser (Spectra-Physics, Berlin, Germany). To avoid photoselection, GUVs must be excited with a circularly polarized beam at the sample plane and imaged at their equatorial plane. A variable wave plate (Newport 5540) located at the input IR port of the microscope was used to compensate for the any polarization effect induced by the microscope optics and this way obtain a circularly polarized beam in the centre of the field of view at the sample plane (Fig. S1). GUVs were imaged through a x63 water-immersion objective (numerical aperture, NA = 1.2) and 512 × 512 pixel images were acquired at 400 Hz per scan-line. The fluorescence emission was collected by non-descanned (NDD) hybrid detectors, as they offer higher sensitivity compared to descanned photomultipliers. The blue edge of the emission spectrum was collected by NDD 1 at 435 ± 20 nm and the red edge by NDD 2 at 500 ± 10 nm. Generalized Polarization (GP) measurements were carried out on unilamellar GUVs.

### Data and Image Analysis

GP images were calculated using in-house developed software based on MATLAB (MathWorks, MA, USA). After smoothing the images with a 2-pixel averaging circular filter and thresholding the intensity, we calculated the GP value for every pixel on the image following equation 1, where *I*_B_ is the intensity collected by NDD 1, *I*_R_ the intensity collected by NDD 2 and *G* is the factor that accounts for the relative sensitivity of the two channels, calibrated with 5 μM Laurdan solution in pure DMSO^56^:


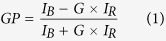


The mean GP value for each lipid mixture was calculated after imaging and analyzing at least 30 GUVs. Two or more independent experiments were routinely carried out and averaged. In the case of co-existing phases in the same vesicle, our software allows the GP to be calculated for each domain separately.

### Planar Supported Phospholipid Layers

Phospholipid monolayers were spread from chloroform/methanol 9:1 (v/v) solutions onto a 5 mM Tris (pH 7.4), 150 mM NaCl subphase, in a thermostated Langmuir-Blodgett trough (NIMA Technologies, Coventry, United Kingdom) as previously described (39, 40). After 10 min to allow for solvent evaporation, monolayers were compressed at 25 cm^2^/min up to the desired pressure and then transferred onto a glass coverslip at 5 mm/min. Specific labeling of fluid disordered phase was attained by including head-group labeled Rho-DOPE (0.5 mol %) in the monolayer composition. Epifluorescence microscopy observation of the planar supported monolayers was performed with a Leica DMI 4000b fluorescence microscope (Leica Microsystems GmbH, Wetzlar, Germany) and a Hamamatsu Orca R-2 digital CCD camera (Hamamatsu Photonics, Japan). AFM images were obtained from the mica-supported films using a JPK Nanowizard II atomic force microscope (JPK Instruments, Germany) operated in contact mode, using silicon nitride tips with a spring constant of 0.1 N/m.

### Cell entry assays

HIV-1 pseudoviruses were produced by transfection of human kidney HEK293T cells with the full-length *env* clone JRCSF (kindly provided by Jamie K. Scott and Naveed Gulzar, Simon Fraser University, BC, Canada) using calcium phosphate. Cells were co-transfected with vectors pWXLP-GFP and pCMV8.91, encoding a green fluorescent protein and an env-deficient HIV-1 genome, respectively (provided by Patricia Villace, CSIC, Madrid, Spain). After 24 h, the medium was replaced with Optimem-Glutamax II (Invitrogen Ltd, Paisley, UK) without serum. Two days after transfection, the pseudovirus particles were harvested, passed through 0.45 μm pore sterile filters (Millex^®^ HV, Millipore NV, Brussels, Belgium) and finally concentrated by ultracentrifugation in a sucrose gradient. HIV entry was determined using TZM-bl target cells (AIDS Research and Reference Reagent Program, Division of AIDS, NIAID, NIH, contributed by J. Kappes). HIV pseudoviruses diluted to a 10–15% tissue culture infectious dose in PBS were deposited onto Poly-L-Lysine-coated 96-well plates, and incubated at 4 °C for 40 minutes. After washing, free poly-lysine was blocked for 20 minutes by medium addition (%90 DMEM, %10 FBS) at 37 °C. Several dilutions of a given virucide in PBS were subsequently applied for 90 min at 37 °C. After three washing steps, 1,1 × 10^4^ TZM-bl target cells were layered on top of immobilized virions in the presence of 30 μg/mL DEAE-dextran (Sigma-Aldrich, St-Louis, MO). Infection levels after 72 hours were inferred from the number of GFP-positive cells as determined by flow cytometry using a BD FACSCalibur Flow Cytometer (Becton Dickinson Immunocytometry Systems, Mountain View, CA).

## Additional Information

**How to cite this article**: Huarte, N. *et al.* Functional organization of the HIV lipid envelope. *Sci. Rep.* **6**, 34190; doi: 10.1038/srep34190 (2016).

## Supplementary Material

Supplementary Information

## References

[b1] Wilen C. B., Tilton J. C. & Doms R. W. HIV: Cell Binding and Entry. Cold Spring Harb Perspect Med 2, doi: 10.1101/cshperspect.a006866 (2012). PMC3405824 22908191

[b2] Checkley M. A., Luttge B. G. & Freed E. O. HIV-1 Envelope Glycoprotein Biosynthesis, Trafficking, and Incorporation. J Mol Biol 410, 582–608, doi: 10.1016/j.jmb.2011.04.042 (2011). 21762802 PMC3139147

[b3] Waheed A. A. & Freed E. O. The Role of Lipids in Retrovirus Replication. Viruses 2, 1146 (2010). 20740061 10.3390/v2051146 PMC2927015

[b4] Lorizate M. & Krausslich H. G. Role of lipids in virus replication. Cold Spring Harb Perspect Biol 3, a004820, doi: 10.1101/cshperspect.a004820 (2011). 21628428 PMC3179339

[b5] Eckert D. M. & Kim P. S. Mechanisms of viral membrane fusion and its inhibition. Annu Rev Biochem 70, 777–810 (2001). 11395423 10.1146/annurev.biochem.70.1.777

[b6] Ward A. B. & Wilson I. A. Insights into the trimeric HIV-1 envelope glycoprotein structure. Trends Biochem Sci 40, 101–107, doi: 10.1016/j.tibs.2014.12.006 (2015). 25600289 PMC4310573

[b7] Sundquist W. I. & Kräusslich H.-G. HIV-1 Assembly, Budding, and Maturation. Cold Spring Harb Perspect Med 2, doi: 10.1101/cshperspect.a006924 (2012). PMC3385941 22762019

[b8] Freed E. O. HIV-1 assembly, release and maturation. Nat Rev Micro 13, 484–496, doi: 10.1038/nrmicro3490 (2015). PMC6936268 26119571

[b9] Brugger B. *et al.* The HIV lipidome: a raft with an unusual composition. Proc Natl Acad Sci USA 103, 2641–2646, doi: 10.1073/pnas.0511136103 (2006). 16481622 PMC1413831

[b10] Chan R. *et al.* Retroviruses human immunodeficiency virus and murine leukemia virus are enriched in phosphoinositides. J Virol 82, 11228–11238, doi: 10.1128/JVI.00981-08 (2008). 18799574 PMC2573248

[b11] Lorizate M. *et al.* Comparative lipidomics analysis of HIV-1 particles and their producer cell membrane in different cell lines. Cell Microbiol 15, 292–304, doi: 10.1111/cmi.12101 (2013). 23279151

[b12] Lorizate M. *et al.* Probing HIV-1 membrane liquid order by Laurdan staining reveals producer cell-dependent differences. J Biol Chem 284, 22238–22247, doi: 10.1074/jbc.M109.029256 (2009). 19553682 PMC2755948

[b13] Callahan M. K. *et al.* Phosphatidylserine on HIV Envelope Is a Cofactor for Infection of Monocytic Cells. J Immunol 170, 4840–4845, doi: 10.4049/jimmunol.170.9.4840 (2003). 12707367

[b14] Soares M. M., King S. W. & Thorpe P. E. Targeting inside-out phosphatidylserine as a therapeutic strategy for viral diseases. Nat Med 14, 1357–1362 (2008). 19029986 10.1038/nm.1885 PMC2597367

[b15] Li M. *et al.* TIM-family proteins inhibit HIV-1 release. Proc Natl Acad Sci USA 111, E3699–E3707, doi: 10.1073/pnas.1404851111 (2014). 25136083 PMC4156686

[b16] Henriques S. T. *et al.* Phosphatidylethanolamine Binding Is a Conserved Feature of Cyclotide-Membrane Interactions. J Biol Chem 287, 33629–33643, doi: 10.1074/jbc.M112.372011 (2012). 22854971 PMC3460461

[b17] Phoenix D. A., Harris F., Mura M. & Dennison S. R. The increasing role of phosphatidylethanolamine as a lipid receptor in the action of host defence peptides. Prog Lipid Res 59, 26–37, doi: 10.1016/j.plipres.2015.02.003 (2015). 25936689

[b18] Hoffmann P. R. *et al.* Interaction between Phosphatidylserine and the Phosphatidylserine Receptor Inhibits Immune Responses *In Vivo*. J Immunol 174, 1393–1404, doi: 10.4049/jimmunol.174.3.1393 (2005). 15661897

[b19] Klymchenko Andrey S. & Kreder R. Fluorescent Probes for Lipid Rafts: From Model Membranes to Living Cells. Chemistry & Biology 21, 97–113, doi: 10.1016/j.chembiol.2013.11.009 (2014). 24361047

[b20] Bagatolli L. A. To see or not to see: lateral organization of biological membranes and fluorescence microscopy. Biochim Biophys Acta 1758, 1541–1556, doi: 10.1016/j.bbamem.2006.05.019 (2006). 16854370

[b21] Scherer E. M., Leaman D. P., Zwick M. B., McMichael A. J. & Burton D. R. Aromatic residues at the edge of the antibody combining site facilitate viral glycoprotein recognition through membrane interactions. Proc Natl Acad Sci USA 107, 1529–1534, doi: 10.1073/pnas.0909680107 (2010). 20080706 PMC2824387

[b22] Alam S. M. *et al.* Role of HIV membrane in neutralization by two broadly neutralizing antibodies. Proc Natl Acad Sci USA 106, 20234–20239, doi: 10.1073/pnas.0908713106 (2009). 19906992 PMC2787149

[b23] Yang S.-T., Kiessling V., Simmons J. A., White J. M. & Tamm L. K. HIV gp41-mediated membrane fusion occurs at edges of cholesterol-rich lipid domains. Nat Chem Biol 11, 424–431, doi: 10.1038/nchembio.1800 (2015). 25915200 PMC4433777

[b24] Carravilla P., Nieva J. L., Goñi F. M., Requejo-Isidro J. & Huarte N. Two-Photon Laurdan Studies of the Ternary Lipid Mixture DOPC:SM:Cholesterol Reveal a Single Liquid Phase at Sphingomyelin:Cholesterol Ratios Lower Than 1. Langmuir 31, 2808–2817, doi: 10.1021/la504251u (2015). 25658036

[b25] Levental I., Grzybek M. & Simons K. Raft domains of variable properties and compositions in plasma membrane vesicles. Proc Natl Acad Sci USA 108, 11411–11416, doi: 10.1073/pnas.1105996108 (2011). 21709267 PMC3136254

[b26] Sezgin E. *et al.* Adaptive Lipid Packing and Bioactivity in Membrane Domains. PLoS One 10, e0123930, doi: 10.1371/journal.pone.0123930 (2015). 25905447 PMC4408024

[b27] Bakht O., Pathak P. & London E. Effect of the structure of lipids favoring disordered domain formation on the stability of cholesterol-containing ordered domains (lipid rafts): identification of multiple raft-stabilization mechanisms. Biophys J 93, 4307–4318, doi: 10.1529/biophysj.107.114967 (2007). 17766350 PMC2098711

[b28] Cruz A. & Pérez-Gil J. Langmuir Films to Determine Lateral Surface Pressure on Lipid Segregation. Methods Mol Biol 400, 439–457, doi: 10.1007/978-1-59745-519-0_29 (2007). 17951751

[b29] Veatch S. L. & Keller S. L. Organization in Lipid Membranes Containing Cholesterol. Phys Rev Lett 89, 268101 (2002). 12484857 10.1103/PhysRevLett.89.268101

[b30] Yuan C. B., Furlong J., Burgos P. & Johnston L. J. The size of lipid rafts: An atomic force microscopy study of ganglioside GM1 domains in sphingomyelin/DOPC/cholesterol membranes. Biophys J 82, 2526–2535 (2002). 11964241 10.1016/S0006-3495(02)75596-3 PMC1302043

[b31] Lawrence J. C., Saslowsky D. E., Edwardson J. M. & Henderson R. M. Real-time analysis of the effects of cholesterol on lipid raft behavior using atomic force microscopy. Biophys J 84, 1827–1832 (2003). 12609884 10.1016/s0006-3495(03)74990-x PMC1302751

[b32] Honerkamp-Smith A. R., Veatch S. L. & Keller S. L. An introduction to critical points for biophysicists; observations of compositional heterogeneity in lipid membranes. Biochim Biophys Acta 1788, 53–63, doi: 10.1016/j.bbamem.2008.09.010 (2009). 18930706 PMC3156111

[b33] Lee D. W. *et al.* Relating domain size distribution to line tension and molecular dipole density in model cytoplasmic myelin lipid monolayers. Proc Natl Acad Sci USA 108, 9425–9430, doi: 10.1073/pnas.1106368108 (2011). 21606329 PMC3111332

[b34] Campbell S. M., Crowe S. M. & Mak J. Virion-associated cholesterol is critical for the maintenance of HIV-1 structure and infectivity. AIDS 16, 2253–2261 (2002). 12441796 10.1097/00002030-200211220-00004

[b35] Guyader M., Kiyokawa E., Abrami L., Turelli P. & Trono D. Role for Human Immunodeficiency Virus Type 1 Membrane Cholesterol in Viral Internalization. J Virol 76, 10356–10364, doi: 10.1128/jvi.76.20.10356-10364.2002 (2002). 12239312 PMC136590

[b36] Apellaniz B., Ivankin A., Nir S., Gidalevitz D. & Nieva J. L. Membrane-proximal external HIV-1 gp41 motif adapted for destabilizing the highly rigid viral envelope. Biophys J 101, 2426–2435, doi: 10.1016/j.bpj.2011.10.005 (2011). 22098741 PMC3218338

[b37] Khaselev N. & Murphy R. C. Structural characterization of oxidized phospholipid products derived from arachidonate-containing plasmenyl glycerophosphocholine. J Lipid Res 41, 564–572 (2000). 10744777

[b38] Liao Z., Graham D. R. & Hildreth J. E. K. Lipid Rafts and HIV Pathogenesis: Virion-Associated Cholesterol Is Required for Fusion and Infection of Susceptible Cells. AIDS Res Hum Retroviruses 19, 675–687, doi: 10.1089/088922203322280900 (2003). 13678470

[b39] Bobardt M. D. *et al.* Hepatitis C virus NS5A anchor peptide disrupts human immunodeficiency virus. Proc Natl Acad Sci USA 105, 5525–5530, doi: 10.1073/pnas.0801388105 (2008). 18378908 PMC2291127

[b40] Badani H., Garry R. F. & Wimley W. C. Peptide entry inhibitors of enveloped viruses: The importance of interfacial hydrophobicity. Biochim Biophys Acta 1838, 2180–2197, doi: 10.1016/j.bbamem.2014.04.015 (2014). 24780375 PMC7094693

[b41] Lenard J., Rabson A. & Vanderoef R. Photodynamic inactivation of infectivity of human immunodeficiency virus and other enveloped viruses using hypericin and rose bengal: inhibition of fusion and syncytia formation. Proc Natl Acad Sci USA 90, 158–162 (1993). 7678335 10.1073/pnas.90.1.158 PMC45619

[b42] Vigant F., Santos N. C. & Lee B. Broad-spectrum antivirals against viral fusion. Nat Rev Micro 13, 426–437, doi: 10.1038/nrmicro3475 (2015). PMC4554337 26075364

[b43] Hollmann A., Castanho Miguel A. R. B., Lee B. & Santos Nuno, C. Singlet oxygen effects on lipid membranes: implications for the mechanism of action of broad-spectrum viral fusion inhibitors. Biochem J 459, 161–170, doi: 10.1042/bj20131058 (2014). 24456301

[b44] Ryu Y.-S. *et al.* Reconstituting ring-rafts in bud-mimicking topography of model membranes. Nat Commun 5, 4507, doi: 10.1038/ncomms5507 (2014). 25058275 PMC4124864

[b45] Apellaniz B. *et al.* Cholesterol-Dependent Membrane Fusion Induced by the gp41 Membrane-Proximal External Region-Transmembrane Domain Connection Suggests a Mechanism for Broad HIV-1 Neutralization. J Virol 88, 13367–13377, doi: 10.1128/jvi.02151-14 (2014). 25210180 PMC4249078

[b46] Volinsky R., Paananen R. & Kinnunen Paavo K. J. Oxidized Phosphatidylcholines Promote Phase Separation of Cholesterol-Sphingomyelin Domains. Biophys J 103, 247–254, doi: 10.1016/j.bpj.2012.06.017 (2012). 22853902 PMC3400785

[b47] Zhu P. *et al.* Distribution and three-dimensional structure of AIDS virus envelope spikes. Nature 441, 847–852, doi: 10.1038/nature04817 (2006). 16728975

[b48] Mattila J.-P. *et al.* A hemi-fission intermediate links two mechanistically distinct stages of membrane fission. Nature 524, 109–113, doi: 10.1038/nature14509 (2015). 26123023 PMC4529379

[b49] Wolf M. C. *et al.* A broad-spectrum antiviral targeting entry of enveloped viruses. Proc Natl Acad Sci USA 107, 3157–3162, doi: 10.1073/pnas.0909587107 (2010). 20133606 PMC2840368

[b50] St.Vincent M. R. *et al.* Rigid amphipathic fusion inhibitors, small molecule antiviral compounds against enveloped viruses. Proc Natl Acad Sci USA 107, 17339–17344, doi: 10.1073/pnas.1010026107 (2010). 20823220 PMC2951442

[b51] Simons K. & Ikonen E. Functional rafts in cell membranes. Nature 387, 569–572, doi: 10.1038/42408 (1997). 9177342

[b52] London E. Membrane fusion: A new role for lipid domains ? Nat Chem Biol 11, 383–384, doi: 10.1038/nchembio.1812 (2015). 25978994

[b53] Chojnacki J. *et al.* Maturation-Dependent HIV-1 Surface Protein Redistribution Revealed by Fluorescence Nanoscopy. Science 338, 524–528, doi: 10.1126/science.1226359 (2012). 23112332

[b54] Brandenberg O. F., Magnus C., Regoes R. R. & Trkola A. The HIV-1 Entry Process: A Stoichiometric View. Trends Microbiol 23, 763–774, doi: 10.1016/j.tim.2015.09.003 (2015). 26541228

[b55] Bligh E. G. & Dyer W. J. A rapid method of total lipid extraction and purification. Can J Biochem Physiol 37, 911–917 (1959). 13671378 10.1139/o59-099

[b56] Gaus K. *et al.* Visualizing lipid structure and raft domains in living cells with two-photon microscopy. Proc Natl Acad Sci USA 100, 15554–15559, doi: 10.1073/pnas.2534386100 (2003). 14673117 PMC307606

[b57] Markosyan R. M., Cohen F. S. & Melikyan G. B. Time-resolved Imaging of HIV-1 Env-mediated Lipid and Content Mixing between a Single Virion and Cell Membrane. Mol Biol Cell 16, 5502–5513, doi: 10.1091/mbc.E05-06-0496 (2005). 16195349 PMC1289397

