# Supplementary material for: Functional organization of the HIV lipid envelope
Source: Sci Rep. 2016 Sep 28;6:34190. doi: 10.1038/srep34190 (PMC5039752; doi:10.1038/srep34190)
Supplement: Supplementary Information [file srep34190-s1.pdf]

Supplementary information

Functional organization of the HIV lipid envelope

Nerea Huarte, Pablo Carravilla, Antonio Cruz, Maier Lorizate, Jon A. Nieto-Garai,

Hans-Georg Kräusslich, Jesús Pérez-Gil, Jose Requejo-Isidro, and José L. Nieva

Table S1: Synthetic virus-like (VL) lipid mixtures used in this work

	PC	CHOL	SM	DHSM	PE	pl-PE	PS
**VL-0**	15 % (DOPC)	45 %	40 %				
**VL-1**	37 % (DOPC)	46 %	17 %				
**VL-2**	14 % (DOPC)	46 %	17 %		16 % (DOPE)		7 % (DOPS)
**VL-3**	14 % (POPC)	46 %	17 %		16 % (POPE)		7 % (POPS)
**VL-4**	14 % (POPC)	46 %	12 %	5 %	3 % (POPE)	13 %	7 % (POPS)

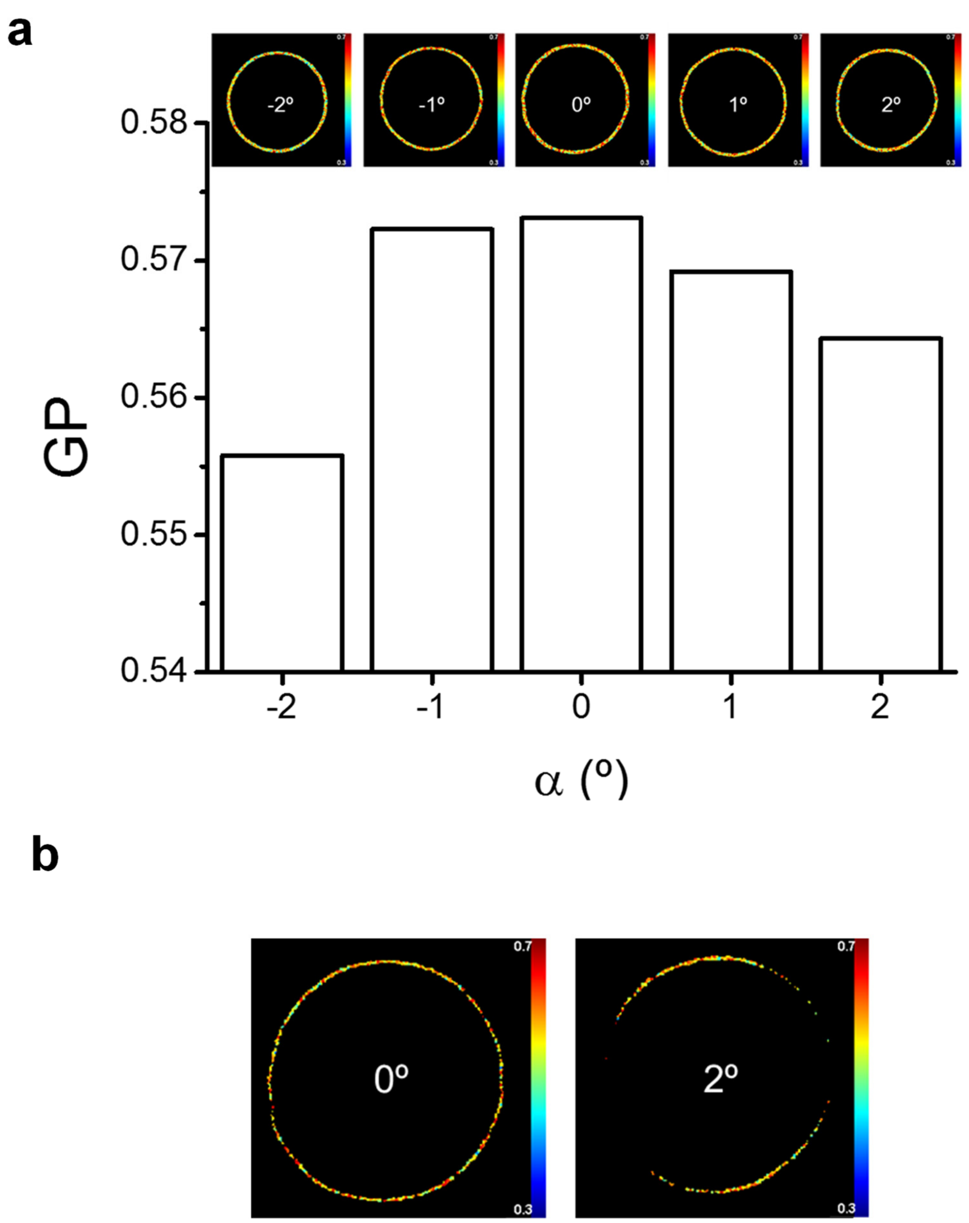

Fig. S1: Quantification of the generalized polarization is very sensitive to small polarization mismatches. Photoselection is the preferential excitation of a subset of fluorophores that are aligned with the polarization axis of the excitation beam. Laurdan's dipole excitation moment is aligned with the lipid acyl chains. To avoid photoselection, spherical GUVs must be excited with a circularly polarized beam at the sample plane and imaged at their equatorial plane. a) Small departures from circular polarization result in

artifactual GP quantification. We calculated the GP values of a VL-2 GUV labeled with 1% Laurdan at small incremental retardance angles. A variable wave plate was used to compensate accurately for the small but non-negligible polarization changes that the excitation beam undergoes as it is relayed through the optics inside the microscope to the sample (see Methods). Circular polarization is achieved at the angle at which the average GP for the whole GUV is maximal and the standard deviation of the GP pixel distribution is minimal, indicating negligible effect of photoselection on the GP. A GP variation of 2.5% was detected even at small departures from the optimal (e.g. -2º or 2º), which, results in the increase of the overall uncertainty of the experiment. b) Photoselection at 2º was not noticeable in GP images at standard analysis conditions, but it became apparent when raw images were threshold to 75% of the maximal intensity. Under these conditions no photoselection can be observed for the GP image on the left, which was therefore chosen as optimal for accurate GP calculation. GP color scale: 0.3 to 0.7.

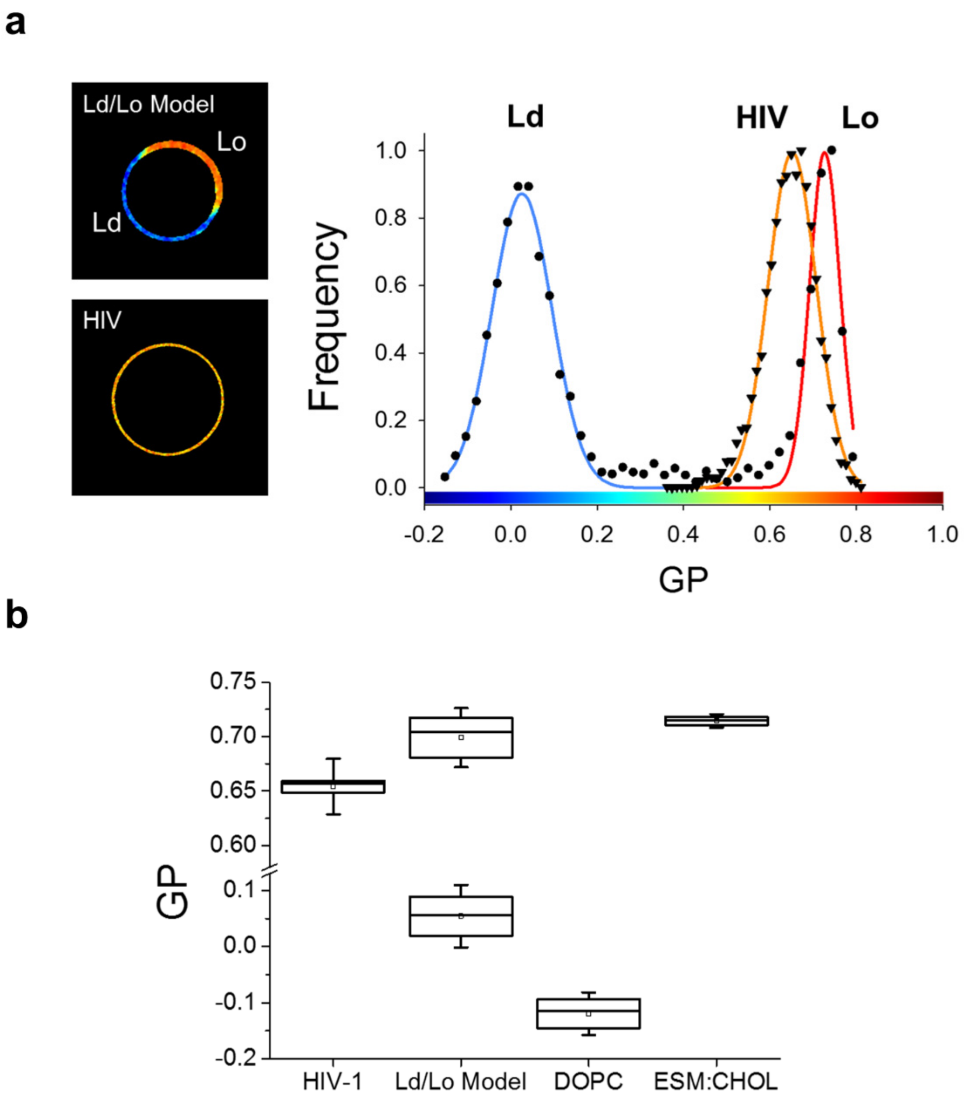

Fig. S2: The viral envelope shows a high degree of lateral packing. a) GP pixel distribution of a representative GUV of the Ld/Lo phase-separated model (DOPC:SM:Chol 2:2:1) (image on the left, top) and a representative GUV reconstituted from HIV membranes (image on the left, bottom). The HIV-reconstituted GUV shows tighter lateral packing than the Ld phase of the Ld/Lo phase-separated model GUV, but less than the Lo phase of the same model. b) Statistical analysis of the GP of a population of GUVs reconstituted from HIV membranes, Ld/Lo phase-separated model (DOPC:SM:CHOL 2:2:1), Ld (DOPC) and Lo (SM:CHOL 1:1) models. The statistical analysis validates the individual GUV behavior shown in previous panel (n > 30, at least three independent repetitions). A Mann-Whitney non-parametric test confirmed that the distributions of all populations are different at the 5% significance level. GP color scale: -0.2 to 0.8.

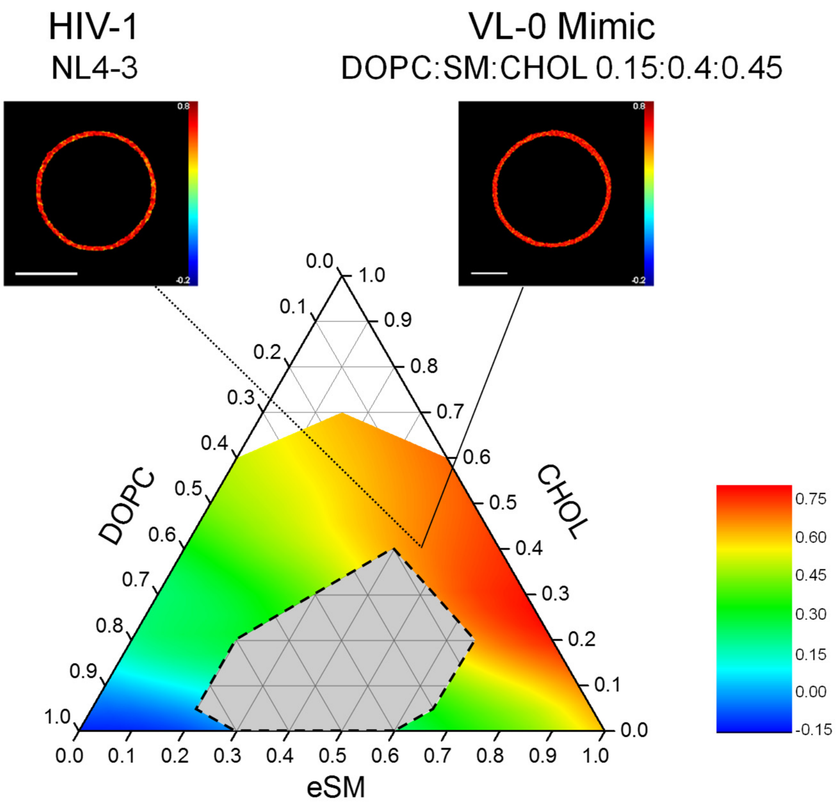

Fig. S3: GP phase diagram of the DOPC:SM:Chol ternary mixture as determined by Carravilla et al. (reference 24). GP values are plotted in false color, red representing the most compact lipid packing and blue the less compact. Lipid composition of the VL-0 packing mimic has been extrapolated from the GP value corresponding to the HIV-reconstituted GUVs.

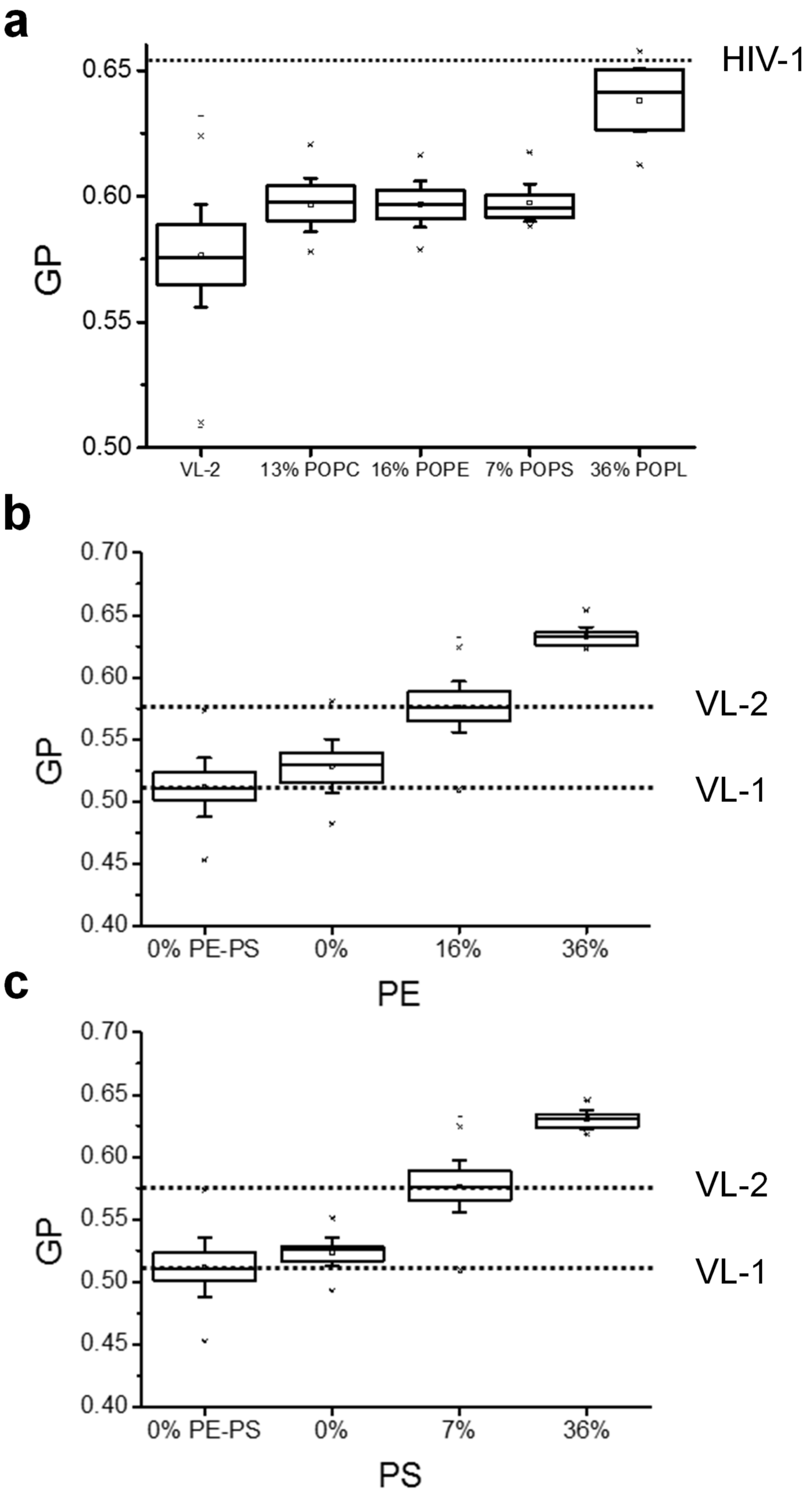

Fig. S4: Contribution of lipid composition to the Laurdan GP values measured for the VL-2 mimic. a) Effect of acyl chain saturation. The change in fatty acid composition from 1, 2-dioleoyl to 1-palmitoyl-2-oleoyl was independently tested for each

glycerophospholipid (POPC, POPE and POPS) and for the combination of the three (POPL). The dotted line indicates the mean value determined for the HIV mixture. b, c) Effect of increasing aminophospholipid content: increasing mol percentages of PE (b) or PS (c) replace PC in the measured samples. Mean GP values determined for VL-1 and VL-2 mixtures are indicated by the dotted lines.

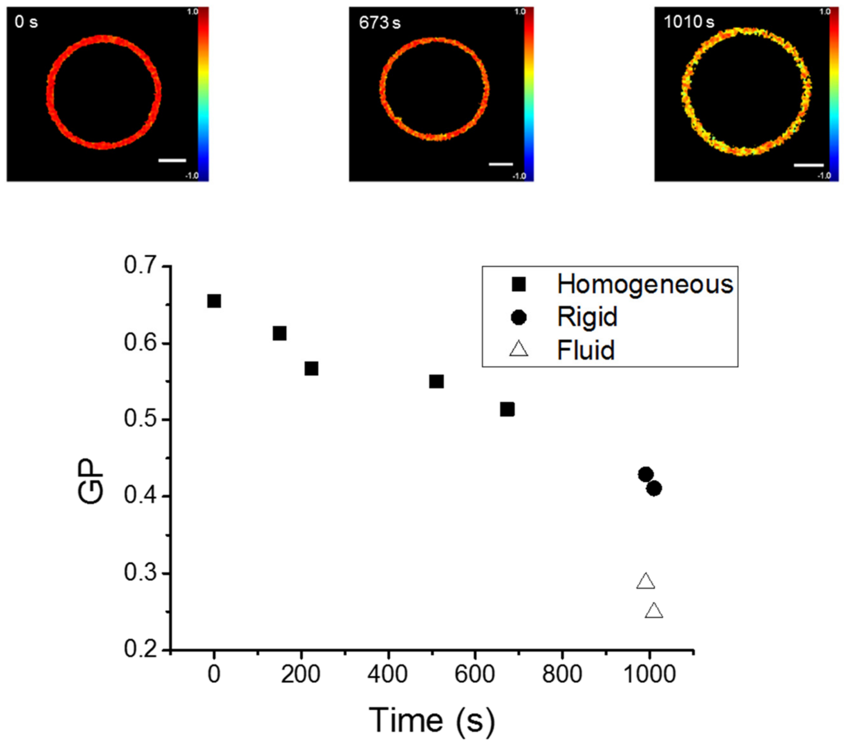

Fig. S5: Time-evolution of the GP of a single GUV reconstituted from HIV membranes upon treatment with M□CD. An individual HIV-GUV was imaged at different times after addition of M□CD. The average GP decreased steadily due to the effect of M□CD. 15 minutes after adding M□CD the GP pixel-distribution of the GUV was best fit to a bimodal distribution with a rigid (top, circle) and a fluid (bottom, triangle) component. GP color scale: -1 to 1.

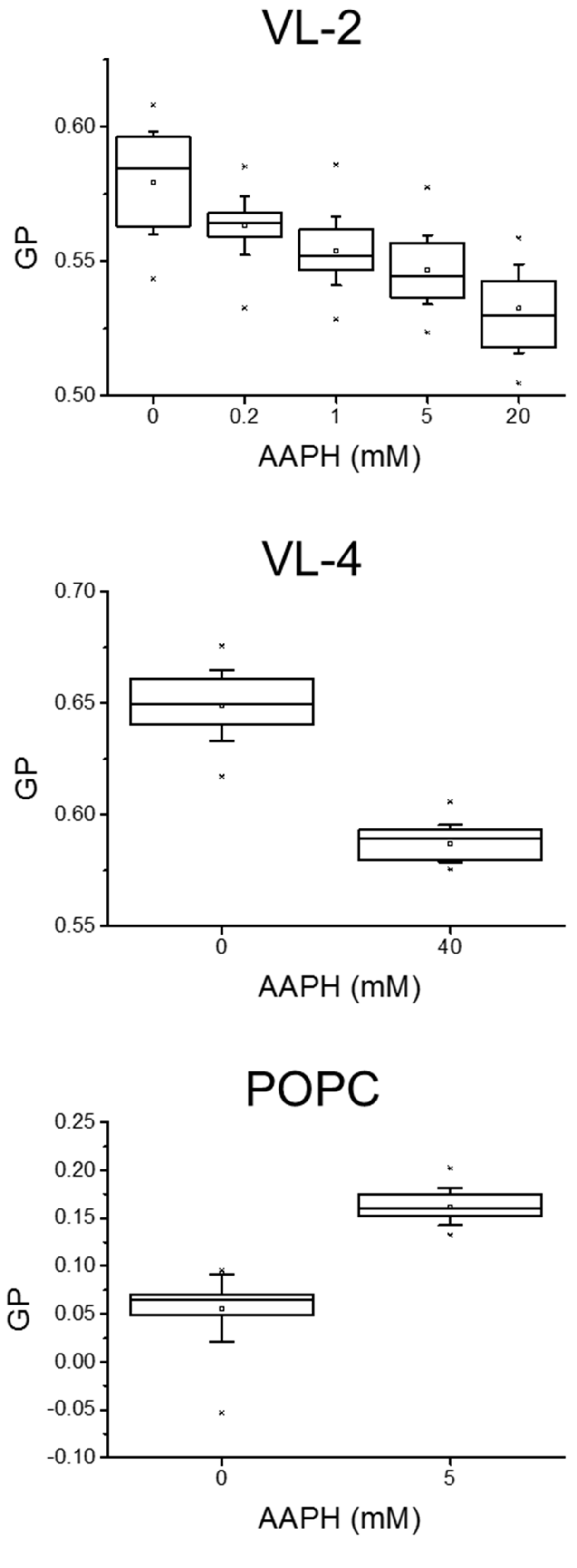

Fig. S6: Laurdan GP values determined for different model membranes incubated with AAPH.

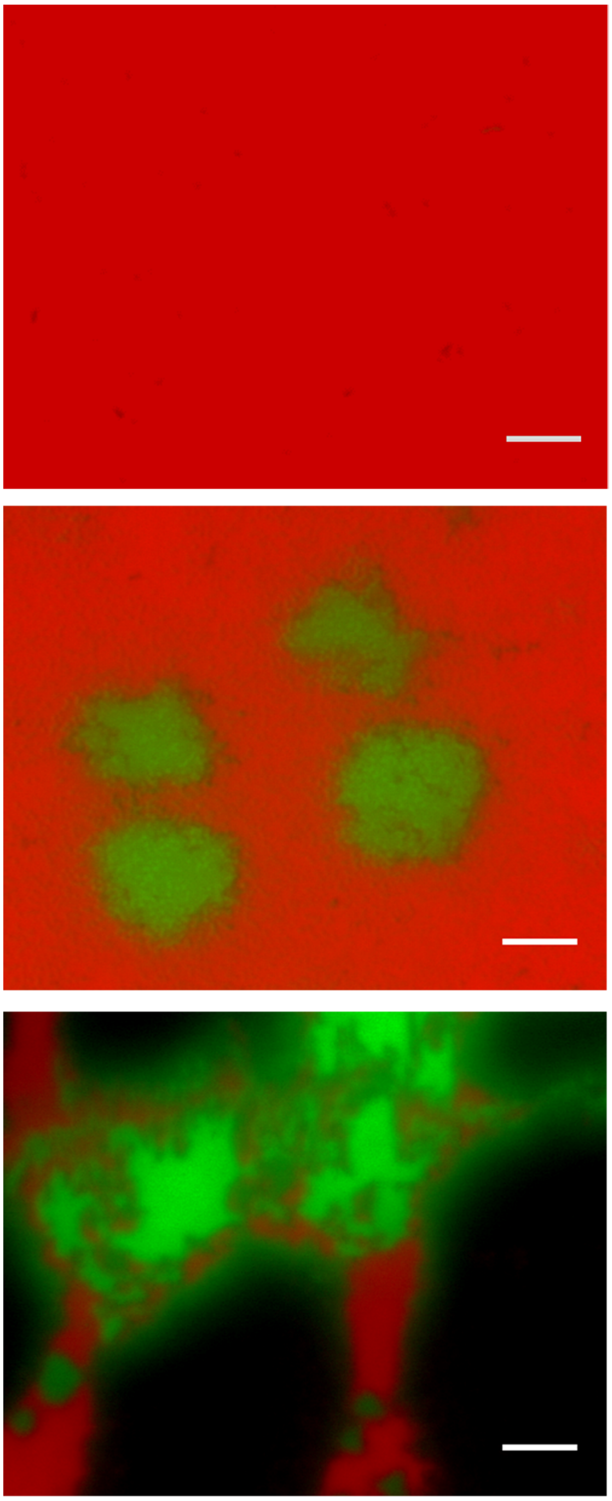

Fig. S7: Lateral reorganization of the HIV monolayer incubated with M□CD. HIV lipids were doped with 0.1 mole % each of the probes Rho-DOPE (red channel) and Topfluor-Chol (green channel). Epifluorescence images were taken for untreated samples (top), and for samples incubated with M□CD (1 mM) for 10 and 30 min (middle and bottom panels, respectively). Conditions otherwise as in Figure 5a.

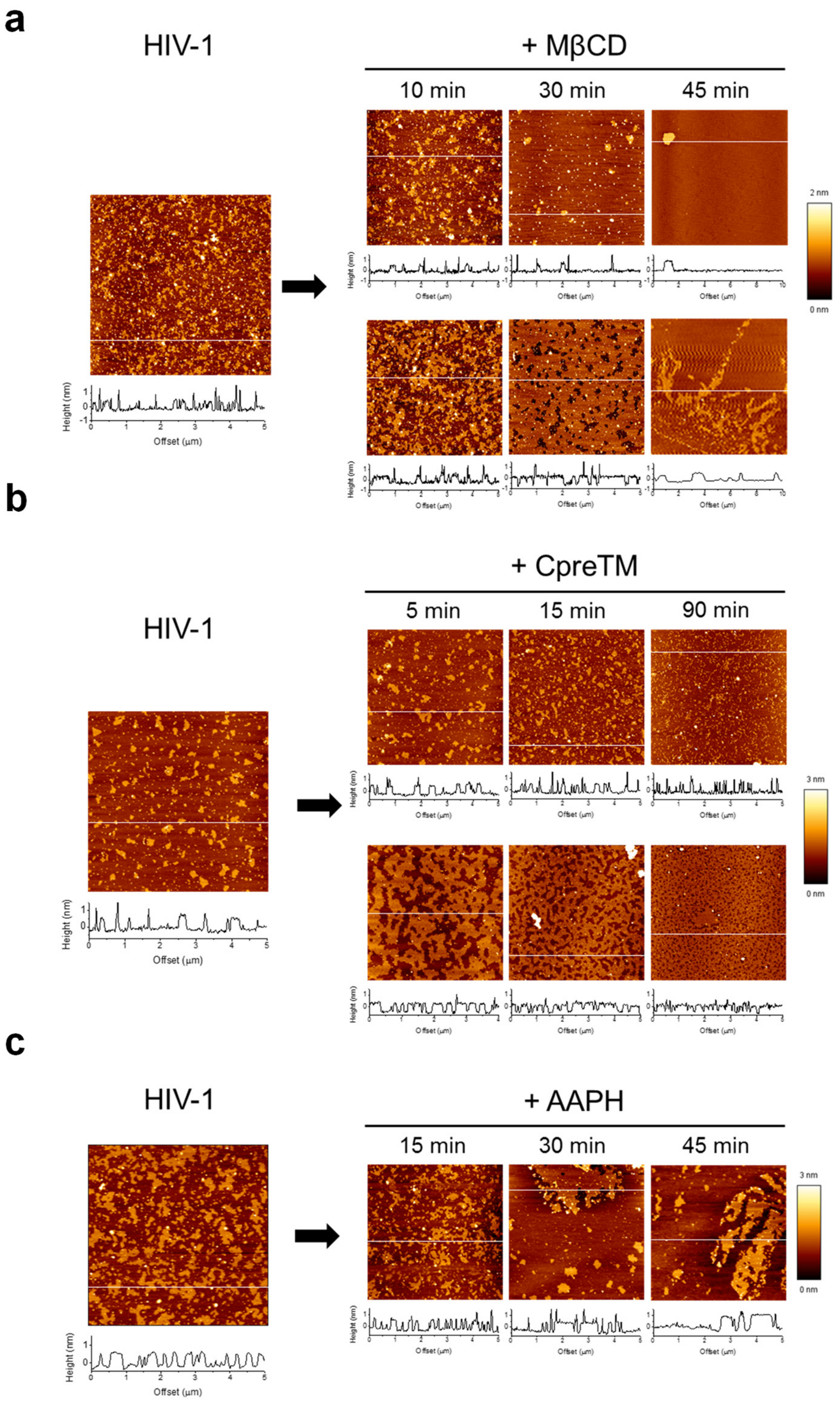

Fig. S8: AFM images and topological profiles of virucide-treated HIV monolayers showing the evolution of laterally de-mixed domains over time. a) Samples treated with M□CD display fluid areas increasingly depleted of rigid domains (top-right). By comparison, rigid areas grow and then shrink by apparently spilling their contents into the

surrounding fluid area (bottom-right). b) CpreTM-treated monolayers display quite unaltered fluid areas (top-right), while rigid domains grow and become more compact over time (bottom-right). c) In monolayers incubated with AAPH, rigid domains cluster into bigger platforms with time, originating mostly bare fluid areas (right panels). Experimental conditions as in Figure 5b.

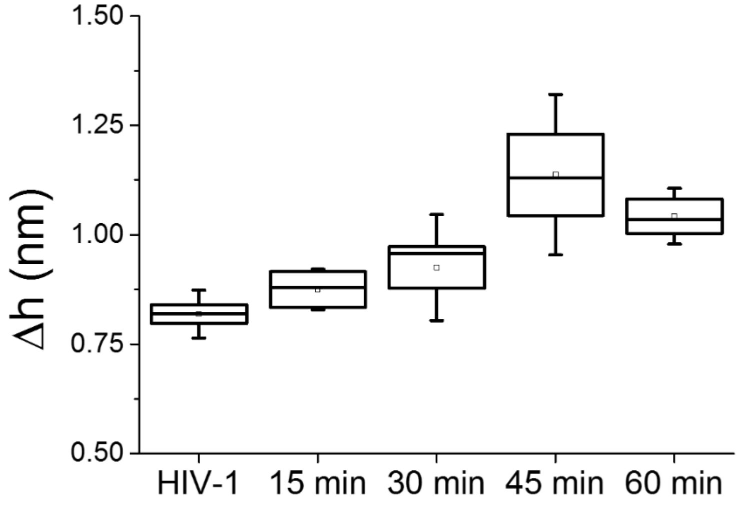

Fig. S9: Increase in height (Δh) with the surrounding membrane of de-mixed rigid domains in a HIV monolayer incubated with AAPH for different times.
